# Acetylcholinesterase Inhibitory Potential of Various Sesquiterpene Analogues for Alzheimer’s Disease Therapy

**DOI:** 10.3390/biom11030350

**Published:** 2021-02-25

**Authors:** Ashwani Arya, Rubal Chahal, Rekha Rao, Md. Habibur Rahman, Deepak Kaushik, Muhammad Furqan Akhtar, Ammara Saleem, Shaden M. A. Khalifa, Hesham R. El-Seedi, Mohamed Kamel, Ghadeer M. Albadrani, Mohamed M. Abdel-Daim, Vineet Mittal

**Affiliations:** 1 Department of Pharmaceutical Sciences, Maharshi Dayanand University, Rohtak 124001, Haryana, India; ashwaniarya5@rediffmail.com (A.A.); imrubal1989@gmail.com (R.C.); deepkaushik1977@gmail.com (D.K.); 2 Department of Pharmaceutical Sciences, Guru Jambeshwar University of Science & Technology, Hisar 125001, Haryana, India; rekhaline@gmail.com; 3 Department of Pharmacy, Southeast University, Banani, Dhaka 1213, Bangladesh; pharmacisthabib@gmail.com; 4 Department of Global Medical Science, Wonju College of Medicine, Yonsei University, Wonju 26384, Korea; 5 Riphah Institute of Pharmaceutical Sciences, Riphah International University, Lahore Campus, Lahore 54000, Pakistan; furqan.pharmacist@gmail.com; 6 Department of Pharmacology, Faculty of Pharmaceutical Sciences, Government College University Faisalabad, Faisalabad 38000, Pakistan; amarafurqan786@hotmail.com; 7 Department of Molecular Biosciences, The Wenner-Gren Institute, Stockholm University, S-106 91 Stockholm, Sweden; 8 Pharmacognosy Group, Department of Medicinal Chemistry, Uppsala University, Biomedical Centre, Box 574, 751 23 Uppsala, Sweden; hesham.el-seedi@farmbio.uu.se; 9 International Research Center for Food Nutrition and Safety, Jiangsu University, Zhenjiang 212013, China; 10 Department of Chemistry, Faculty of Science, Menoufia University, Shebin El-Kom 32512, Egypt; 11 Department of Medicine and Infectious Diseases, Faculty of Veterinary Medicine, Cairo University, Giza 12211, Egypt; m_salah@cu.edu.eg; 12 Department of Biology, College of Science, Princess Nourah bint Abdulrahman University, Riyadh 11474, Saudi Arabia; gmalbadrani@pnu.edu.sa; 13 Pharmacology Department, Faculty of Veterinary Medicine, Suez Canal University, Ismailia 41522, Egypt

**Keywords:** cognition, coumarins, lactones, phytochemicals, neurodegeneration, acetylcholinesterase, Alzheimer’s disease

## Abstract

Alzheimer’s disease (AD) is a gradually growing irreversible illness of the brain that almost affects every fifth person (aged > 80 years) in the world. World Health Organization (WHO) also revealed that the prevalence of this disease will enhance (upto double) significantly upto 2030. The poor cholinergic transmission at the synapse is considered to be one of the main reasons behind the progression and occurrence of this disorder. Natural inhibitors of acetylcholine (ACh) such as galanthamine and rivastigmine are used commercially in the treatmentof AD. The biomolecules such assesquiterpenes, possess a great structural diversity and are responsible for a plethora of pharmacological properties. The potential of various sesquiterpenes as anticholinesterase has been reviewed in this article. For this purpose, the various databases, mainly PubMed, Scopus, and Web of Science were investigatedwith different keywords such as “sesquiterpenes+acetylcholinesterase” and “sesquiterpenes+cholinesterase+inhibitors” in the surveyed time frame (2010–2020). A vast literature was evident in the last decade, which affirms the potential of various sesquiterpenes in the improvement of cholinergic transmission by inhibiting the AChE. After data analysis, it was found that 12 compounds out of a total of 58 sesquiterpenes were reported to possess IC_50_ < 9 μM and can be considered as potential candidates for the improvement of learning and memory. Sesquiterpene is an important category of terpenoids, found to possess a large spectrum of biological activities. The outcome of the review clearly states that sesquiterpenes (such as amberboin, lipidiol, etc.) from herbs could offer fresh, functional compounds for possible prevention and treatment of AD.

## 1. Introduction

**Figure 1:**
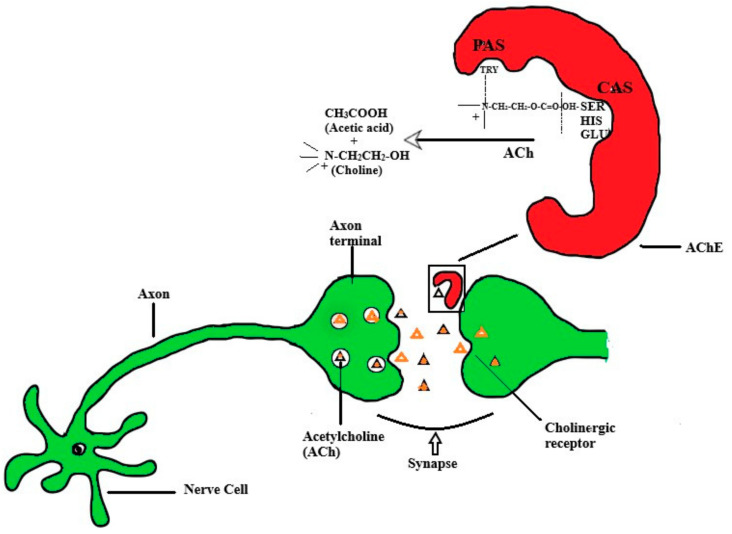
Acetylcholine (Ach) transmission through synapse (cholinergic transmission) and action of acetylcholinesterase (AChE) on acetylcholine. ACh: acetylcholine; AChE: acetylcholinesterase; PAS: peripheral anionic site; CAS:catalytic active site; TRY: tryptophan; SER: serine; HIS: histidine; GLU: glutamate.

Alzheimer’s disease (AD), the most leading kind of dementia, is an age-linked neurodegenerative disease affecting approximately 24 million people around the world [1,2,3]. The World Health Organization (WHO) has estimated that there will be more than 100% growth of this number by the end of the year 2030. Moreover, as per the data available, it can be stated that AD affects almost every fifth person at the age of 80 years and up [4,5]. AD is a gradually growing irreversible illness of the brain and is characterized by the loss of memory, language impairment, disorientation, abstract thinking impairment, mood swings, behavior changes, loss of initiative, and trouble in performing the regular tasks on a daily basis [6,7]. The formation and deposition of the β-amyloid (Aβ) plaques and accumulation of intracellular hyperphosphorylated tau proteins or neurofibrillary tangles are the main hallmarks of the AD pathophysiology [8,9,10,11,12]. In addition, the inflammation of neurons, stress due to free radicals, and mitochondrial dysfunction are involved in neuron degeneration and result in poor cholinergic transmission [13,14,15]. It has also been observed that cholinergic neurons are highly damaged in the forebrain during the progression of AD [16,17]. An increase of acetylcholine concentration (ACh) at the synapse by inhibiting the acetylcholinesterase (AChE) is one of the approaches to slow down the progression of AD [18,19,20,21,22,23,24,25,26]. AChE is assumed to lyse the acetylcholine at a very fast rate (up to 10,000 molecules per second). The enzyme mainly has two active sites, peripheral anionic site (PAS) and catalytic active site (CAS), for the binding of substrate, i.e., acetylcholine. The ACh temporarily sequesters to the PAS through π-cation interaction between tryptophan on enzyme surface and quaternary amine of ACh. Later, a tetrahedral intermediate forms at CAS (at the deeper gorge of enzyme) by a reaction between the oxygen atom of the acetyl group of ACh and serine–histidine–glutamate residue of the enzyme through a covalent bond (Figure 1) [27,28]. PAS of AChE also reported to have an affinity toward the peptide of Aβ and the binding of this protein to enzyme promotes the amyloid fibrogenesis and converts the non-amyloidogenic form of Aβ to amyloidogenic form [29]. Moreover, the conformational changes due to AChE-Aβ complex formation also enhanced the metabolism of Aβ and senile plaque generation [30].

Cholinesterase inhibitors (ChEi) such as galantamine and rivastigmine mainly bind to the peripheral site in a competitive and reversible manner and spare the ACh at the synapse and thus help in the improvement of cholinergic transmission [31,32]. Rivastigmine can also hydrolyze the butyrylcholinesterase (BuChE) present at glial cells of the temporal cortex region of the brain [33]. AChE inhibition can help in maintaining the normal neurotransmission mediated by ACh in a healthy brain and could prevent the occurrence or slow down the progression of AD. However, during the late stages of AD, the concentration of AChE decreases significantly (upto 45%) and BuChE is enhanced (upto 40–90%). Thus, ChEi, which are effective against both enzymes, are highly desirable in AD therapy [34]. Moreover, the reduction in substrate concentration (AChE) by these inhibitors may affect the Aβ plaque formation and thus justify the use of ChEi as multi-target therapy for AD. Current drugs available for the treatment of the disease if given timely, at their best may delay the related fatal alterations but are evidently incapable of reversing the neurodegeneration process associated.

For a long time, plants have been seen as a healthcare reserve and are being used in treating and preventing human diseases and ailments. In an exploration of novel biologically active natural compounds, many traditionally used medicinal plants are being screened and evaluated for their pharmacological activities [35]. Several medicinal plants or herbs have been used to improve cognitive function, memory, and in treating neurodegenerative diseases such as AD in alternative systems of medicine [36,37,38,39]. Preclinical and clinical studies witnessed the effectiveness of the natural compounds isolated from the plants for the neuroprotective consequences through various invitro and invivo methods [40,41]. Furthermore, the phytoconstituents, which occur naturally, play a crucial role in treating some kind of aging-related illness [42,43,44,45,46,47,48]. Much research has been devoted to sesquiterpenes that own long history of usage as antimicrobial, antibacterial, anti-inflammatory, cytotoxic, antiviral, antifungal, antiulcer, and anti-allergenic agents [49,50,51,52,53,54,55,56,57]. Sesquiterpenes are considered significant for human use and play a significant role in biological systems. [58,59,60,61]. Sesquiterpene such as nerolidol was reported to reduce the damage to biomolecules (DNA, protein, lipids) due to elevated levels of reactive oxygen species in cells [62]. In addition, the cytotoxicity of neuroblastoma cells by Aβ deposition decreased significantly by sesquiterpene lactones [63]. Moreover, the active constituents of different essential oils such as farnesol, caryophyllene, and α-humulene significantly alter the various intracellular signaling parameters of inflammation [64].

Sesquiterpenes are colorless, lipophilic terpenes found naturally in both plants and insects, holding a backbone of 15 carbons (M.F. = C_15_H_24_) with great structure diversity because of the unique and specific layering of various substituents and other functional groups around structural scaffolds [65,66,67]. Naturally, they occur as hydrocarbons or as oxygenated forms comprising the lactones, aldehydes, alcohols, acids, ketones, etc. Furthermore, sesquiterpenes also involve some essential oils and aromatic constituents with various pharmacological activities [68,69,70]. 

In this review article, we provide updated information about various applications of the different sesquiterpenes isolated from various plants and their role as memory enhancers by inhibiting the acetylcholinesterase (AChE).

## 2. Review Methodology and Current Developments

Herbal compounds, owning a standing as the efficient agents in numerous biological systems, currently are drawing interest for inhibiting acetylcholinesterase (AChE) activity or in the prevention of AD. The secondary metabolites of the various classes such as alkaloids, terpenoids, coumarins, flavonoids, etc. have been reported to have potent AChEinhibitory activity [71,72,73,74]. Moreover, as a biologically important class, sesquiterpenes has emerged as a potent inhibitor of AChE in the last decade. The potential of various sesquiterpenes as anticholinesterase has been reviewed in this article. For this purpose, the various databases, mainly PubMed, Scopus, and Web of Science were investigated with different keywords such as “sesquiterpenes+acetylcholinesterase” and “sesquiterpenes+cholinesterase+inhibitors” in the surveyed time frame (2010–2020) [75]. The primary screening of a plethora of plant species for their anti-Alzheimer potential could be carried out by estimating the IC_50_ for AChE using Ellman’s method [76,77,78,79,80]. In the following, the role of sesquiterpenoids of different classes such as lactones, coumarins, agarofuran, and alkaloids, has been described for the possible prevention of AD.

### 2.1. Sesquiterpenoids

Sesquiterpenoids are a group of various natural compounds and are the derivatives of a 15-C precursor called farnesyl pyrophosphate (FPP). Sesquiterpenoids are mainly confined to specialized secretory cells, laticifers, but when there is some biotic stress, and these can also be found in the vacuoles of other cells too. This diverse group is considered vital for the identity and protection of plants (response to allelopathic stimulation) and imparts various biological activities such as antimicrobial and anti-inflammatory effects [35,81,82,83,84]. Moreover, these compounds (Figure 1) can be used to hit pharmacological targets in managing the condition of AD.

Choi et al. [85] isolated a novel seco-illudoid sesquiterpene, pterosinone(**1**) from the ethanolic extract of *Pteridium aquilinum*. Pterosinone was reported to have mild inhibitory activity against acetyl and butyl cholinesterase enzymes with IC_50_ = 88 μM and 73 μM, respectively. It was concluded from the results that sesquiterpenoids could be useful in treating AD. Chougouo et al. [51] isolated artemisinin (**2**) and chrysosplenetin (**3**) from the ethanol extract of *Artemisia annua* Linn. for the modulation of anticholinesterase (AChE) activity. The crude extract with artemisinin and chrysosplenetin showed the inhibition of AChE activity by 72% and 80% at 0.1 mg/mL with IC_50_ of 104 µM and 73 µM.This study supported the possible use of these two compounds in neurological disorders including AD. 

Jung et al. [86] isolated the Valenc-1(10), 3(4),11,(12)-trien-2-one (**4**) from the heartwood extract of *Juniperus chinensis* Linn. This compound exhibited significant butyrylcholinesterase activity with the IC_50_ = 68 μM when compared to the positive control, berberine (IC_50_ = 19 μM), and this property might be attributed to its structural configuration where two double bonds at C_1–10_ and C_3–4_ and C_11–12_with a ketone group at C_2_ are supposed to increase the inhibitory activity. 

Yang et al. [87] reported the isolation of eremophilane sesquiterpenes from the *Aquilaria sinensis* (Lour.) Glig and elucidated their structures by the spectroscopic procedures. Isolated compounds were found out to be 7-β-*H*-9-(10)-ene-11,12-epoxy-8-oxoeremophilane (**5**) (48 ± 2% inhibition at 50 mg/mL with IC_50_ = 275 ± 5 µM); 7α-*H*-9(10)-ene-11,12-epoxy-8-oxoeremophilane (**6**) with 31 ± 1% inhibition at 50 mg/mL with IC_50_ = 491 ± 4 µM); neopetasane (**7**) (62 ± 1% inhibition at 50 mg/mL with IC_50_ = 158 ± 4 µM). 

Chen et al. [88] extracted the sesquiterpenoids from the root of *Valeriana officinalis* var. latifolia and investigated their AChE inhibitory activities by the modified in vitro Ellman method. It was observed that the Volvalerenic acid K (**8**) improved the learning and memory abilities of mice, with IC_50_ = 0.16 µM in vitro. The results of the study showed that, in mice (APPswe/PS1E9 double-transgenic mouse) from middle and high dose groups, the AChT (acetylcholine transferase) activity had been improved in the brain tissues, while the AChE activity was significantly decreased causing an increase in the learning memory.

Moreover, Shi et al. [89] extracted some bioactive sesquiterpenes, oxyphyllanene A, teuhetenone A, oxyphyllol B, and nootkatone from the chloroform extract of *Alpinia oxyphylla* fruits and evaluated for their cognitive potential. Long term exposure to chloroform extract improved the cognitive abilities while performing the behavioral tasks, increased the glutathione peroxidase (GSH-px) activities, decreased the acetylcholinesterase (AChE), malondialdehyde level (MDA), and β-amyloid (Aβ), and ultimately overturned the microglia activation, neuronal acidophilia degeneration, and cortex and hippocampus nuclear condensation. It was concluded that the *Alpinia oxyphylla* ameliorates the learning and memory deficits by attenuating the oxidative stress, regulating the activation of the microglia, and degeneration of the neuronal acidophilia to reinforce the cholinergic functions. Thus, the bioactive sesquiterpenes isolated from the chloroform extract of *Alpinia oxyphylla* may provide some therapeutic targets for the prevention and cure of AD. In addition, the ethanolic extracts of this plant have shown agood inhibitory activity of 44.49% against AChE at 0.1 mg/mL concentration.

The essential oils containing sesquiterpenoids in comparison to the oils containing monoterpenoids were found to be more effective inhibitors of AChE activity, and the same is the case of mixtures where it ruled by monoterpenoids showed weaker inhibition than the mixture dominated by sesquiterpenoids. Fujiwara et al. [90] evaluated the essential oil obtained from the bark of *Peltophorum dasyrachis* Kurz ex Bakar and the main sesquiterpenoids found were, (+)(*S*)-ar-turmerone (**9**) and (+)-(*S*)-dihydro-ar-turmerone (**10**). These two isolated compounds have been assessed for their AChE inhibitory activity and proved to be potent in performing their action against AChE in a dose-dependent manner. Di hydro derivative of turmerone is most potent with IC_50_ at 82 ± 0.2 μM, followed by volatile oil (IC_50_ = 83 ± 3 μg/mL). Hence, it was concluded from the study that the different compounds isolated from the *Peltophorum dasyrachis* oil are effective and useful in the treatment of AD. Furthermore, the bisabolane-type sesquiterpenoid derivatives from plants, such as (+)-(7*S*,9*S*)-ar-turmerol, (+)-(7*S*,9*R*)-ar-turmerol, (+)-(7*S*,9*S*)-dihydro-ar-turmerol, (+)-(7*S*,9*R*)-dihydro-ar-turmerol, (+)-(*S*)-ar-curcumene, and (+)-(*S*)-dihydro-ar-curcumene, were synthesized which were found to exhibit the acetylcholinesterase inhibitory activity in the order hydrocarbons <alcohols < ketones. The maximum activity was observed through the oxidation of the functional group at C_9_ position and C_10_–C_11_ structure of single-bond moiety, (+)-(*S*)-dihydro-ar-turmerone. Moreover, the Turmerone was analyzed to be a competitive inhibitor of AChE, whereas the dihydro derivative is the non-competitive inhibitor of this enzyme. Miyazawa et al. [91] investigated the leaf and stem oil of *Gynura bicolor* DC for AChE inhibitory potential. The leaf oil inhibited the AChE with IC_50_ = 85 µg/mL), On the other hand, stem oil showed the same inhibition with IC_50_ = 92 μg/mL. It indicated the leaf oil as a stronger inhibitor of AChE activity than the stem oil. GC–MS analysis confirmed the presence of (*E*)-β-caryophyllene (31%) and bicycle germacrene (8%) as the major sesquiterpenoids from the *Gynura bicolor* DC leaves oil. The sesquiterpenoids in essential oil from the *Gynura bicolor* were found to act synergistically against AChE inhibition. Christianahet al. [92] extracted the volatile oil from the leaves of *Plectranthus aegyptiacus* (Forssk.) and identified thirty compounds including some sesquiterpenoids such as copaene, α-caryophyllen, germacrene-d, and α-cadinol with a respective concentration of 5%, 6%, 12%, and 8%. The oil was observed to exhibit AChE inhibitory activity with an IC_50_ = 8 ± 0.6 mg/mL, which can be helpful in the treatment of AD. 

Rahali et al. [93] studied the essential oil obtained from flower buds of *Hertia cheirifolia* and showed significant antioxidant activity along with the efficient acetylcholinesterase enzyme inhibitory activity for the prevention of AD. It was concluded that essential oil from flower buds of *Hertia cheirifolia* are rich in the anti-AChE activity of 1.86 mg Eq donepezil/g of essential oil due to the presence of Germacrene d.

Similarly, the greenish-yellow color essential oil from the *Salvia chionantha* moderate AChE and BChE activity, i.e., 57 ± 2% and 41 ± 9%, respectively. Cholinesterase inhibitory activity could be attributed to the presence of Germacrene d, a sesquiterpenoid in the oil. In contrast, the extract exhibited 63 ± 0.8% activity only against BChE at the concentration of 500 μg/mL. The results indicated the moderate potential of essential oil in the prevention of AD [94]. Moreover, the hydroalcoholic extract and the essential oil obtained from *Acorus calamus* (AC) rhizomes for in vitro acetylcholinesterase inhibitory activity by using Ellman’s method. The IC_50_ values for alcoholic extract, essential oil, and two chief constituents of oil, i.e., β-asarone (**11**) and α-asarone (**12**) were reported as 182 ± 17 μg/mL, 11 ± 0.8 μg/mL, 3 ± 0.02 μM, and 46 ± 3 μM, respectively. It was detected that both the compounds had AChE inhibitory activity when physostigmine was taken as the standard inhibitor with an IC_50_ value of 0.3 ± 0.02 µM. The β-asarone showed the maximum inhibition against AChE [95].

Olawuni et al. [96] extracted the essential oils of *Monodora myristica* and *Piper nigrum* Linn. seeds and evaluated them for their anti-cholinesterase and antioxidant activities. Both the oils showed significant AChE and BChE inhibitory activity (*p* < 0.05) in a dose-dependent manner at the concentration of 416 µg/mL. *M. myristica* elicited stronger inhibition for AChE and BChE with IC_50_ of 205 ± 0.06 µg/mL and 178 ± 0.02 µg/mL with 79% and 89% inhibition, whereas the *P. nigrum* oil inhibited the AChE (75%) and BChE (85%) with IC_50_ = 0.3 ± 0.02 µg/mL and 223 ± 0.002 µg/mL, respectively. The various sesquiterpenoids such as phellandrene (18.13%), caryophyllene (4.55%), and copaene (2.23%) present in these oils and may be responsible for the acetylcholinesterase inhibitory potential and could be investigated in future individually. 

Furthermore, the molecular docking study could also be helpful in determining the AChE inhibitory potential of a compound or essential oil. Autodock 4.2 and iGemDock 2.1 software were employed to find out the affinity of isolated essential oil from *Myrciaria floribunda* (H. Westex Wild) for AD target protein AChE. The oil exhibited the AChE inhibitory potential with IC_50_ = 0.08 μg/mL, and this activity is attributed to the higher binding affinity of δ-cadinene and γ-cadinene (oil constituents) toward AChE as predicted by the docking score of –7.35 and –6.77, respectively [97].

**Figure 2:**
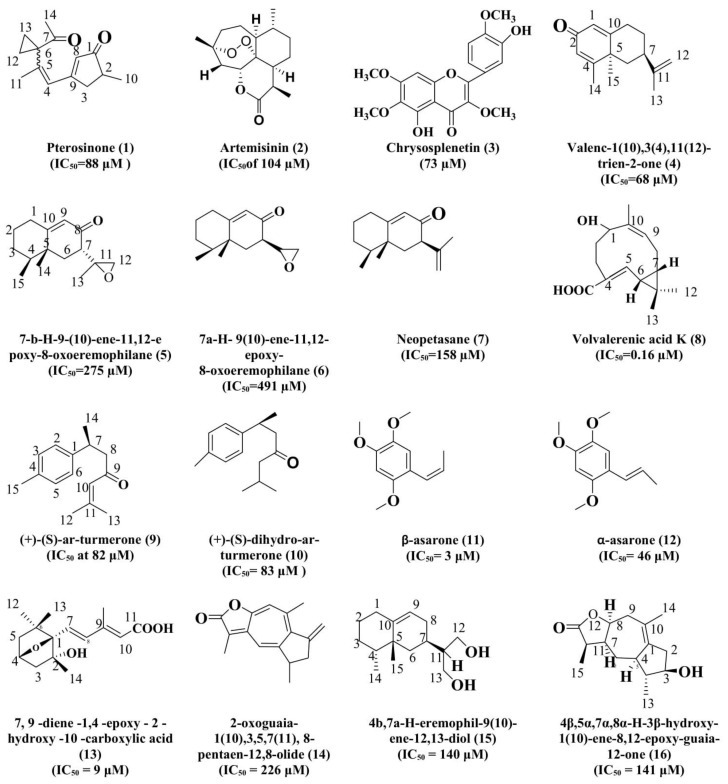

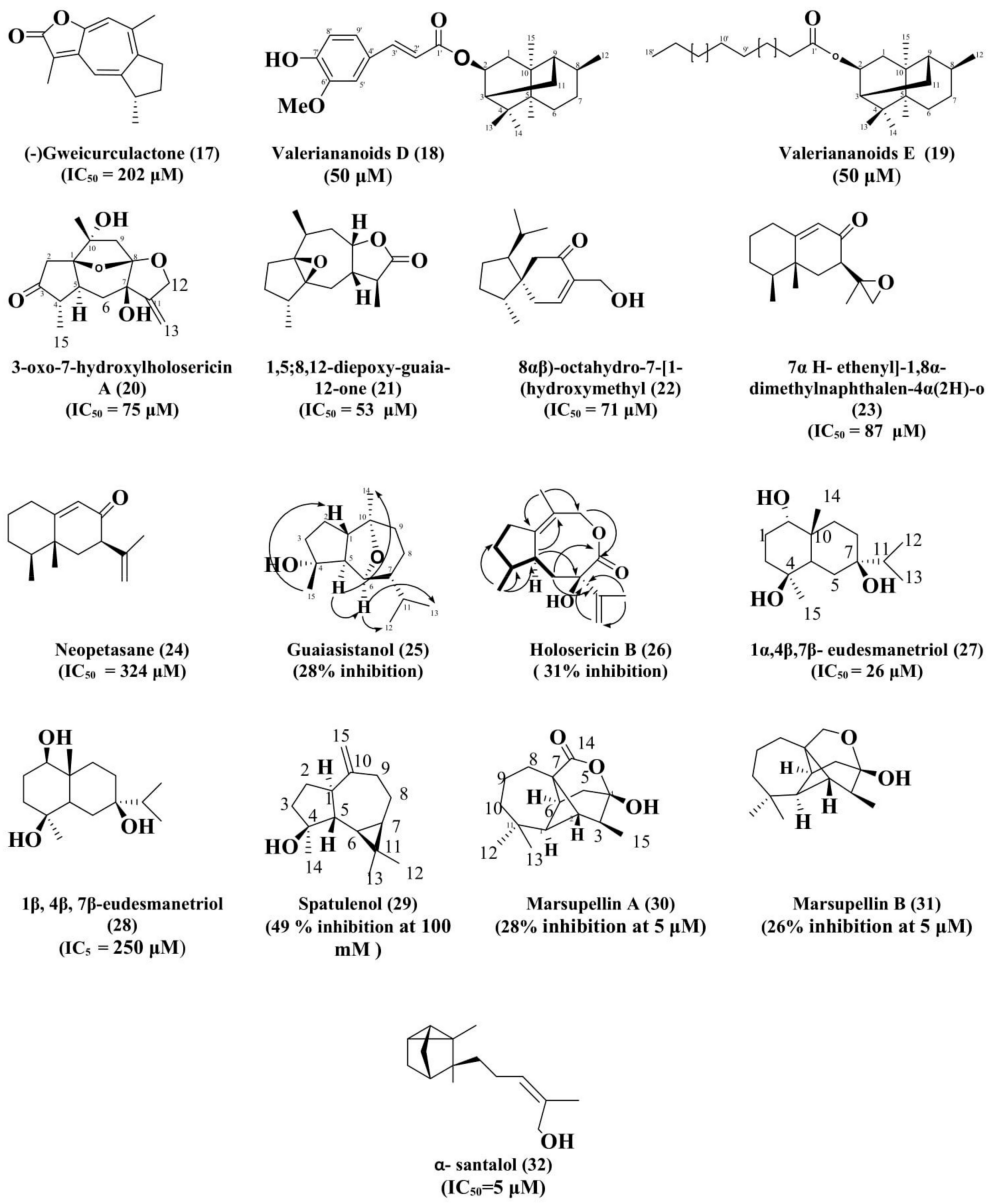
Various sesquiterpenoids moieties with AChE/BChE inhibitory potential.

Some sesquiterpenes isolated from the plants and their worthy roles in the prevention of AD are also summarized in Table 1. The structure of major sesquiterpenoids with AChE inhibitory potential is presented in Figure 2. As the AChE inhibition is one of the treatment approaches for most neurodegenerative diseases, including Alzheimer’sdisease, the essential oils that are easily inhalable and possess the AChE inhibitory potential might present a fresh vision toward the treatment of these neuronal diseases, including AD. 

### 2.2. Sesquiterpene Lactones (SLs) 

The sesquiterpene lactones (SLs) are biosynthesized in the medicinal plants by the oxidative transformation and cyclization of three isoprene units [1]. They constitute the largest and structurally most diverse group of secondary metabolites among several other natural compounds. SLs revealed the potential in nurturing humans as a dietary source and for health, as pharmaceutical agents, because of their efficiency in treating various types of ailments. SLs are formed through the mevalonic acid pathway of the plants. A general characteristic of SLs is the occurrence of the γ-lactone ring, which contains the α-methylene group. The key combinations of the α-methylene-γ-lactone comprise a few ester groups [35,116,117,118,119,120]. SLs are reputable across the plant kingdom that widespread in the families such as Asteraceae, Cactaceae, Euphorbiaceae, Solanaceae, Apiaceae, Acanthaceae, Amaranthaceae, Polygonaceae, Aristolochiaceae, Burseraceae, Illiciaceae, Magnoliaceae, Menispermaceae, Coriariaceae, Chloranthaceae, Lamiaceae, Lauraceae, and Winteraceae [49,50,55]. They are recognized for holding a wide variety of pharmacological and biological activities such as anti-fungal, anti-bacterial, anti-microbial, cytotoxic, antiviral and anti-inflammatory, etc. Moreover, according to folk remedies, SLs are active ingredients to treat influenza, inflammation, diarrhea, burns, and neuron degradation [70,117].

They are frequently isolated from the plants of the Asteraceae/Compositae family and reported to possess significant therapeutic activity. Elsebaia et al. [121] reported on amberboin (**33**) and lipidiol (**34**) as sesquiterpene lactones from the *Volutaria abyssinica* A. Rich (Family-Asteraceae) for the acetylcholinesterase inhibitory activity. Docking study of both compounds confirms the affinity of them towards the active sites of AChE, which indicated inhibitory activity against AChE at IC_50_ = 0.8 ± 0.03 µM and 0.5 ± 0.01 µM, respectively. Further, amberboin also inhibited the BChE at IC_50_ of 0.6 ± 0.13 µM. The results clearly stated that the two isolated sesquiterpene lactones are the better inhibitors of AChE and BChEas compared to the reference, galanthamine (IC_50_ = 3 ± 0.2 μM and 47 ± 1 μM for AChE and BChE, respectively). In addition, the leaves of *Cynara cornigera* (Wild artichoke) from the Asteraceae family were extracted using methanol and seven SLs were isolated. Furthermore, a computational pharmacophore interpretation and docking design were accomplished to assess the pharmacophoric sorts and binding conformation of the isolated compounds in the AChE active site. Out of the seven metabolites, sibthorpine (**35**) showedAChE inhibitory activity presenting this compound to be tested for anti-neurodegenerative activity. It was observed from the study that sibthorpine isolated from *Cynara cornigera* is responsible for the inhibition of anticholinesterase (IC_50_ = 71 μM) activity [122].

Hajimehdipooret al. [123] extracted the SLs from the *Inula oculus-christi* and *I. aucheriana* (Family-Compositae) and evaluated their capability of inhibiting AChE activity by using the Ellman assay method. It was observed that out of the three evaluated compounds, the Gaillardin (**36**) was the most versatile secondary metabolites (IC_50_ for AChE 729 μM) present in the plants and can be investigated for further AD studies.

Ibrahim et al. [124] isolated four sesquiterpene lactones from *Amberboa ramosa* (Family-Asteraceae) using the ethyl acetate soluble fraction and tested them against AChE and BChE. Their IC_50_ (μM) for AChE inhibition was reported as 17 ± 0.01, 9 ± 0.2, 0.9 ± 0.02 and 1 ± 0.08 for sesquiterpene lactones Amberin (**37**), Amberbin-A (**38**), Amberbin-B (**39**), and Amberbin-C (**40**), respectively, and for BChE inhibition was at 3 ± 0.02 (Amberin), 5 ± 0.2 (Amberin-A), 2.5 ± 0.2 (Amberin-B) and 18 ± 0.05 (Amberin-C). This study showed that all the SLs. isolated from *Amberboa ramosa* might be effective against AChE and BChE enzymes and could be used as remarkable lead molecules in the drug development for the prevention of AD. Patel and Amin [125] extracted the 7-hydroxy frullanolide (**41**), a sesquiterpene lactone, from the flower heads of *Sphaeranthus indicus* (Family-Asteraceae) and carried out in vitro anticholinesterase activity of several extracts. Petroleum ether fraction was found to be loaded with SLs (about 80%), accounting for its anti-AChE activity. It was observed that petroleum ether fraction was the only portion with AChE inhibitory potential with an IC_50_ of 37 mg/mL and the extract at a dose of 10 mg/kg, per orally. in mice considerably inverted the cognitive damages caused through the passive avoidance test (*p* < 0.05). 

**Figure 3:**
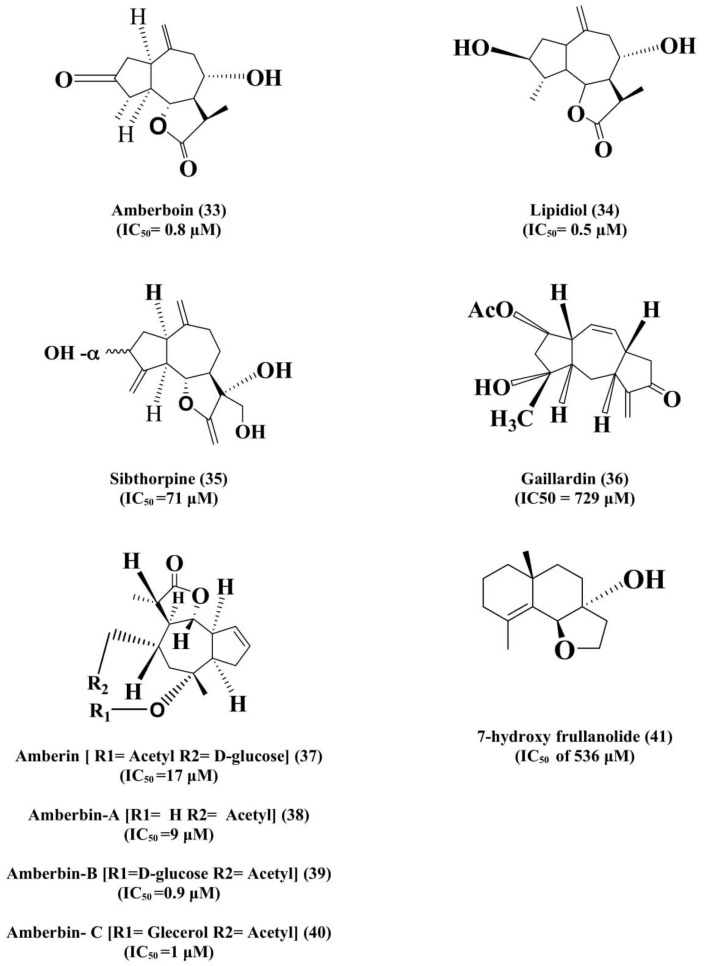
Sesquiterpene lactones/their derivatives for AChE inhibition.

In the light of the above-cited examples, various SLs (Figure 3) seem to be promising acetylcholinesterase inhibitors and helpful in improving the equilibrium between the excitatory and inhibitory potential of neurons and thus can be used in the possible management of AD.

### 2.3. Miscellaneous Sesquiterpenes

The role of sesquiterpenes of different categories such as coumarins, agarofuran, and alkaloids in the AD are described as follows:

#### 2.3.1. Sesquiterpene Coumarins

Sesquiterpene coumarins (SC) is an interesting family of natural products, isolated mainly from *Ferula* genus in which through an ether linkage coumarin moiety mainly umbelliferone (7-hydroxycoumarin) and sometimes isofraxidin (7-hydroxy-6,8-dimethoxycoumarin) or scopoletin (7-hydroxy-6-methoxycoumarin) is connected to the C_15_ terpene moiety. The prenyl-furocoumarin-type sesquiterpenoids represent a different cluster in sesquiterpene coumarins, whereby the involvement of C_3_, coumarin, and C_15_ moiety are connected through the C–C bond. SC is set up in numerous medicinal plants belonging to various other families such as Asteraceae, Rutaceae, Apiaceae, etc. [44,126,127,128]. *Ferula* genus always has been a matter of study and controversy because of its wide chemical variation in different types of plants from different locations. For illustration purposes, investigations of *Ferulacommunis* population from Sardinia revealed that two chemotypes of the plant from this region have different characteristics. One of the chemotypes contained prenylated 4-hydroxycoumarins, which are toxic while the second one demonstrated therapeutic activity because of the presence of the SC ethers [127,128]. 

Guvenalpet al. [129] isolated the sesquiterpene coumarins such as umbelliprenin (**42**) and feselol (**43**) from the chloroform extract of *Heptapteracilicica* fruits. The compounds showed potential inhibitory activity against AChE (IC_50_ = 6 ± 0.03 μM and 1 ± 0.01 μM, respectively) and BChE (IC_50_ = 10 ± 0.24μM and 1 ± 0.19 μM, respectively) and established the plant as an emerging source in AD treatment. Other studies have also been conducted in the past, supporting the anticholinesterase activity of these compounds. 

#### 2.3.2. Dihydro-β-AgarofuranSesquiterpenes

Tricyclic 5,11-epoxy-5-β,10-α-eudesman-4-(14)-ene is the basic moiety accounting for this structurally diverse class, dihydro-β-agarofuransesquiterpenes. These compounds mainly belong to the family Celastraceae and are held responsible for many pharmacological activities such as their anti-HIV, cytotoxic, insecticidal, anti-feedant, and immunosuppressive properties, etc. A lot of therapeutic significance of medicinal plants has been recognized from the family of more oxygenated sesquiterpenoids, which are depending on a tricyclic dihydroagarofuran skeleton type. Studies conducted on the agarofuran compounds with a motive of developing some new AChE inhibitors have achieved great success in the past years and suggested that agarofuran compounds may act as lead compounds in the designing of new, less toxic, and highly selective anticholinesterase agents along with some other positive therapeutic effects [130,131]. 

Alacronet al. [131] isolated constituents with agarofuran skeleton (epoxyeudesmane) from the *Maytenusdisticha* and *Euonymus japonicas* aerial parts and seeds. All dihydro agarofuranoid sesquiterpenes such as, 1-α,6-β,8-α-Triacetoxy-9-β-furoyloxy-β-agarofuran (**44**), 1-α-Hydroxy-6-β,8-α-diacetoxy-9-β-furoyloxy-β-agarofuran (**45**), 1-α,6-β-Diacetoxy-8-α hydroxy-9-β-furoyloxy-β-agarofuran (**46**), 1-α-Acetoxy-6-β,8-α-dihydroxy-9-β-furoyloxy-β agarofuran (**47**), 1-α,2-α,6-β,8-α,15-Pentaacetoxy-9-β-benzoyloxy-β-agarofuran(**48**), 1-α,2-α,3 β,15-Tetraacetoxy-6-β,9-β-dibenzoyl-8-oxo-β-agarofuran(**49**), 1-α,6-β,15-Triacetoxy-9-benzoyloxy-β-agarofuran (**50**), 2-α,3-β,6-β,8-α,15-Pentaacetoxy-1-α,9-β-benzoyloxy-β-agarofuran (**51**) were found to exhibit anti-acetylcholinesterase (AChE) activity. The compounds isolated from seeds of *M. disticha* and *E. japonicas* showed the AChE inhibitory activity with the IC_50_ (mg/mL) of 0.1 ± 0.01 (**44**), 0.3 ± 0.02 (**45**), 0.1 ± 0.004 (**46**), 0.1 ± 0.006 (**47**), 0.1 ± 0.002 (**48**), 0.3 ± 0.015 (**49**), 0.4 ± 0.006 (**50**) and 0.4 ± 0.009 for the compound (**51**). Based on these data, the majority of the compounds indicated acceptable inhibitory activity in the submicromolar concentrations ranges, i.e., 0.1–0.4 mg/mL on comparison with the reference drug, galanthamine. On the other hand, these compounds are found to be selective inhibitors for AChE activity. Alarcon et al. [132] extracted the dihydro-β-agarofuran sesquiterpene from the *Maytenus disticha* (aerial parts) and *Maytenus boaria* (seeds) of Celastraceae family. The compounds and their AChE inhibitory potential of (IC_50_ in mg/mL) were found to be, 1-*α*,2-*α*,6,8*α*-tetraacetoxy-9-benzoyloxy-15-hydroxy agarofuran (**52**) (0.1 ± 0.01), 1-*α*,2-*α*,6-β-triacetoxy-9-β-benzoyloxy-15-hydroxy-β agarofuran (**53**) (0.1 ± 0.01), 1-*α*,2-*α*,6-β-triacetoxy-9-β-benzoyloxy-8-*α*,15-dihydroxy-β-agarofuran (**54**) (0.3 ± 0.02), 1-*α*,2-*α*,6-β,8-*α*,15-pentaacetoxy-9-β-benzoyloxy-β-agarofuran (**55**) (0.2 ± 0.003), 1-*α*-acetoxy-6-β, 9-β-difuroyloxy-4-β-hydroxy- β-agarofuran (**56**) (0.4 ± 0.04), and 6-β,8-*α*-diacetoxy-9-β-furoyloxy-1-*α*-hydroxy-β-agarofuran(**57**) (0.3 ± 0.02). Therefore, it was observed that the agarofuran compounds isolated from the aerial parts of *Maytenus disticha* and seeds of *Maytenus boaria* have good signs for their further use in the treatment of AD or as acetylcholinesterase inhibitors. 

It was concluded from the above-cited examples that the selective inhibitory potential of agarofuran compounds for AChE and low toxicity might lead to the development of novel therapeutic agents for possible AD management.

#### 2.3.3. Sesquiterpene Alkaloids

In the last decades, investigations to find out some natural sources for acetylcholinesterase inhibitory activity have been fastened, and the good thing is that the scientists are achieving progressive results as most of the alkaloids are capable ofimproving cognitive deficits. Among the various constituents that evolved with AChE inhibitory activity, alkaloids type sesquiterpenes have snatched a crowning position [38]. Some of the accessible information about sesquiterpene alkaloids, presented them as the lead molecules of the future prevention of AD, as pinpointed here [133,134]. 

Gul et al. [133] extracted a broad neuroprotective sesquiterpene alkaloid, Huperzine-A (**58**) from the Chinese herb, *Huperia serrata*. This natural compound showed reversible AChE inhibitory activity (IC_50_ = 22 μM) and can enhance cognitive abilities and task switching functions rapidly in people suffering from AD. One more study suggested that this compound may be beneficial in treating hypoxic-ischemic encephalopathy in neonates. Huperzine-A has been proved to lessen the glutamate-induced cytotoxicity by its antagonizing action on cerebral N-methyl-D-aspartate (NMDA) receptors, and this mechanism of antagonism is one plausible justification for its neuroprotection property [134,135,136,137]. Coupled with these studies, Huang et al. [3] investigation about the compound provided a marked base for the establishment of this compound as a reversible, selective, potent, and finely tolerated inhibitor of acetylcholinesterase which can considerably improve the memory and learning deficit in the persons suffering from Alzheimer’s disease. 

**Figure 4:**
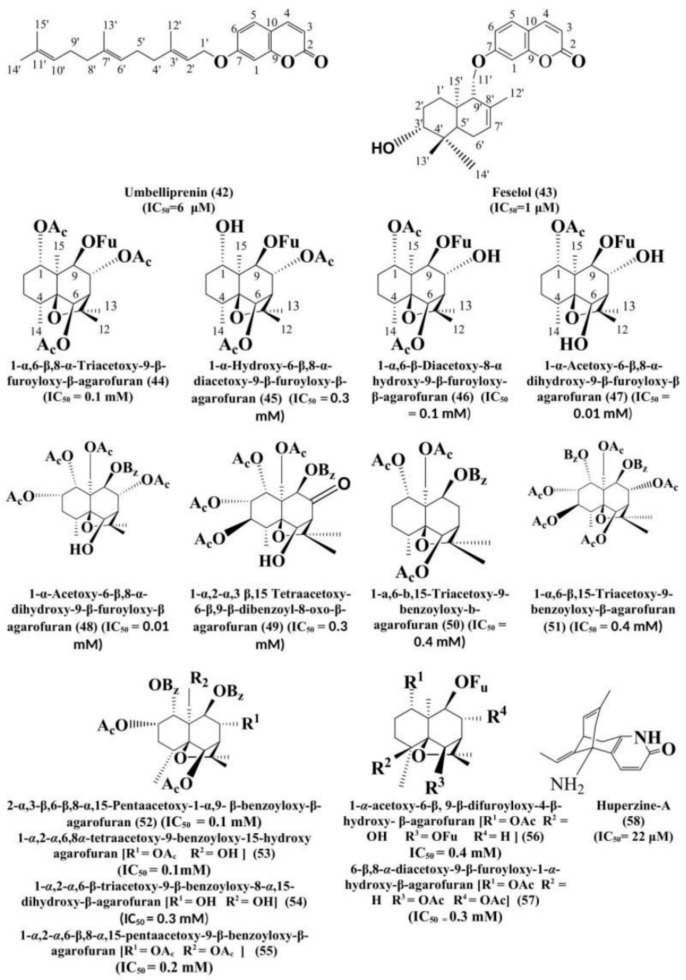
Structure of miscellaneous sesquiterpenes found effective against acetylcholinesterase.

The above discussed sesquiterpenes, from plants or natural sources, have been demonstrated as potent inhibitors of acetylcholinesterase. Figure 4 indicates the structure of miscellaneous sesquiterpenes found effective against acetylcholinesterase. 

## 3. Sesquiterpenes for Alternate AD Targets

The pathophysiology of AD is very much complicated and a single target therapy is not beneficial for the management of this neurodegenerative disease. The various sesquiterpenes could also be employed for the multiple targets such as reduction in Aβ plaque, neuroinflammation, oxidative stress and alteration in GABAergic transmission.

Qi et al. [138] investigated the waste produced of *Stigma maydis* (maize) industry and isolated macrocarpene type sesquiterpenes compounds, namely Stigmene A and Stigmene B, and screened for their Aβ aggregation inhibitory activities. It was observed that (%) decrease in Aβ aggregation was found more in the case of Stigmene A (82.9 ± 2.52%) and Stigmene B (70.1 ± 3.01%) when compared against a positive control, (69.8 ± 1.55%). The result of the study suggested that the presence of these two valuable compounds made *Stigma maydis* crop waste a promising source for the pharmaceutical firms involved in the manufacturing/research of formulations for neurological disorders such as AD. 

Huangaet al. [139] studied the memory-enhancing property of the *Ginkgo biloba* (Family-Ginkgoaceae) leaf extract. The chief constituent of the extract, bilobalide, a sesquiterpene trilactone, was found to deter the straight action of gamma-aminobutyric acid (GABA) receptors. Dueto this property, the constituent tends to have a vibrant role in treating cognitive dysfunction in dementia. The bilobalide IC_50_ (4.6 ± 0.5 μM) was reported as comparable to conventional antagonists of GABA receptors, picrotoxinin (2.4 ± 0.5 μM) and bicuculline (2.0 ± 0.1 μM) at α_1_β_2_γ_2L_GABA_A_ receptors against GABA neurotransmitter. Bilobalide amplified the levels of GABA in the cerebral cortex and hippocampus of mice; in addition, bilobalide also enhanced the neuronal excitability in hippocampus sites through the blockade of GABAergic neurotransmission, which is directly correlated with the learning and memory motions. The potency level of bilobalide decreased with increasing GABA concentrations indicating a module of competitive antagonism. 

Amoahet al. [140] studied the SLs and sesquiterpene alcohol (SA) isolated from the *Hedyosmum brasiliense* (Family–Chloranthaceae) for anti-inflammatory and anti-oxidative activities. During the investigation, it was observed that aromadendrane-4β,10α-diol (ARD), 13-hydroxy-8,9-dehydroshizukanolide (HDS) and podoandin (PDA), efficiently ameliorated the Aβ peptide-induced memory impairment in models of the passive avoidance task (*p* < 0.05). The above-stated SLs compounds showed a considerable effect on oxidative stress and enhanced the memory power in animals that received Aβ-42 infusion. However, the neuroprotective effects possessed by these tested compounds were found more correlated to the existence of the guaiane ring in comparison to the presence of the lactone ring for which mechanism of action is described by Michael-type nucleophilic addition. This study confirmed that ARD, HDS, and PDA, the constituents of *H. brasiliense,* are efficient in inhibiting the cognitive deficits of animals and may be used in treating AD.

Neuroinflammation is one of the major factors of AD and other disorders related to cholinergic transmission. It can be attenuated by reducing the expression of NF-κB-p65 via NLRP3 pathways. Thus targeting the transcription factor (NF-κβp65) could be the novel approach for the treatment of AD. This factor upregulates the expression of inflammatory cytokines and genes related to oxidative stress and BACE-1 (B-site APP Cleaving Enzyme), responsible for the production of β-amyloid [141,142]. Wanga et al. [143] isolated the nootkatone from petroleum ether extract of *Alpiniae oxyphyllae* Fructus and demonstrated that it could improve the lipopolysaccharide-induced memory and learning impairment, which was proposed to be associated with its attenuating neuroinflammatory activity through the expression of transcription factor NF-κB-p65. Nootkatone at 10 mg/kg showed significant improvement in neuroprotective potential on evaluation through various behavioral models (Morris water maze and Y maze). This study indicated that the compound, nootkatone, might play a role as a potent therapeutic agent in treating AD and neuroinflammation via improvement in cholinergic transmission, clearing amyloid-β-peptide, and reducing oxidative stress.

In addition, ambrosin, another sesquiterpene lactone, was isolated from the ethanolic extract of *Ambrosia maritima* (Family-Asteraceae). Although it was not evaluated for the AChE inhibitory potential but found to reduce the expression of NF-κβp65, transcription factor, in experimental mice. Halting the expression of this factor could reduce the production of β-amyloid (Aβ), which results in enhancement of cholinergic transmission at the synapse. Moreover, ambrosin at 10 mg/kg dose also reduces the detrimental effect of lipopolysaccharide on the learning and memory potential of mice [144]. 

Hence ambrosin and nootkatone, as novel sesquiterpenes, could significantly reduce the expression of this transcription factor (NF-κβp65) in experimental groups.

The Jatanolides, Jatamansone, and Jatamansic acid are the main sesquiterpene alkaloids isolated from the *Nardostachys jatamansi,* which are helpful in cognitive disorders as they improve the learning and memory supremacy of a person [145,146]. Joshi and Parle [146] studied the ethanolic extract of *Nardostachys jatmansi* in mice to evaluate its memory enhancing property and found it capable of significantly improving the learning and memory at a dose of 200 mg/kg and also reversed the scopolamine (0.4 mg/kg, i.p.) and diazepam (1 mg/kg, i.p.) induced amnesia. It was concluded that memory enhancement could be a result of the facilitation of cholinergic transmission in the brain becausethe extract showed potential against scopolamine-induced impairment. Furthermore, the extract also reversed the aging-related amnesia proving it as a beneficial memory restorative constituent in elderly individuals with dementia. The mechanism of action can be credited to its anti-oxidative property. The neuroprotective, anti-cerebral ischemic and antioxidative properties of various Jatanolides present in the plant may be the reason for its memory-enhancing activity. 

The above-cited compounds may potentially be used as moieties in the building of a better multi-target anti-AD drug for possible prevention and treatment of AD.

## 4. Market Formulations to Alleviate AD Symptoms

There are various formulations available in the market that claim to alleviate the symptoms of AD. However, in reality, only a few marketed drugs clinically improve cholinergic transmission by inhibiting the cholinesterase enzyme. Moreover, the literature review reveals that only a single cholinesterase inhibitor is in Phase II and Phase III clinical trials, for AD management [147,148]. Moreover, Huperzine A is the only sesquiterpene alkaloid that is clinically established as an AChE inhibitor and receivedthe approval of USFDA to manage AD. In addition, the large number of formulations having extracts of *Ginkgo biloba* and *Nardostachys jatamansi* are mentioned in Table 2 and mainly prescribed as a nutraceutical for delaying symptoms (such as amnesia) of AD. The extracts of these herbs are reported to contain sesquiterpenes such asBilabolide and Jatanolides/Jatamansone as main ingredients. Preclinical studies prove the neuroprotective role of these herbs due to reduction in neuronal damage by free radicals and restoration of calcium homeostasis [41]. Hence, this could be the possible rationale for the inclusion of these herbs as food supplements for AD.

## 5. Final Remarks and Future Prospects

**Figure 5:**
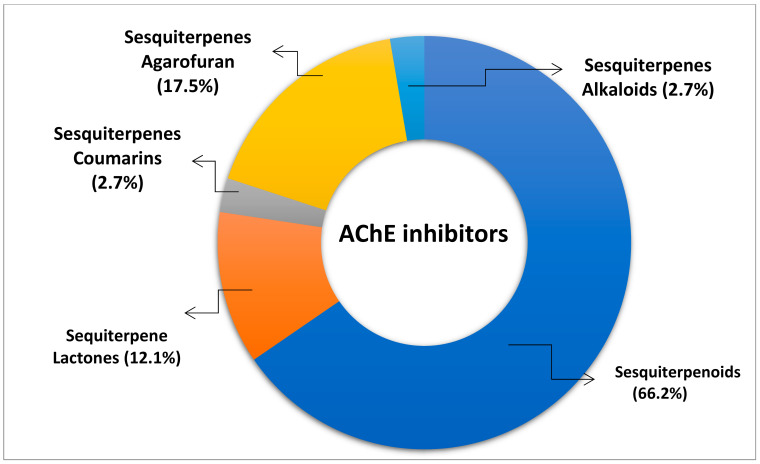
Percentage of various categories of sesquiterpenes with AChE inhibitory potential.

Sesquiterpene is an important category of terpenoids, found to possess a large spectrum of biological activities. As discussed in the present review, these plant actives have been reported to play a significant role in the improvement of cholinergic transmission by inhibiting the AChE. In literature, multiple sesquiterpenes havebeen evident from the past decade to possess the AChE inhibitory activity. The percentage of compounds having anti acetylcholinesterase potential from different categories of sesquiterpenes has been indicated in Figure 5. 

Critically analyzing the literature, it has been found that 12 compounds out of total 58 sesquiterpenes were reported to possess IC_50_ < 9 µM and can be considered as potential candidates for the improvement of learning and memory. However, mere possession of significant IC_50_ (for AChE) should not be the criteria for the selection of a compound for anti-Alzheimer potential. Because the evaluation of cholinesterase inhibition potential of compounds, with carbonyl group, by in vitro methods such as Ellman reagent could produce the false positive [149]. Hence, they should be further evaluated in animal models for final confirmation of results.

In the light of reviewed articles, it can also be stated that the Asteraceae/Compositae family plants reported yielding the sesquiterpenes lactones such as amberboin and lipidiol that were having very low inhibitory concentration (IC_50_ = 0.8 μM and 0.5 μM, respectively) against acetylcholinesterase. Hence, the herbs from this family could be further explored for plant actives with significant anti-Alzheimer potential. 

Furthermore, the pathophysiology of Alzheimer’s disease includes the degeneration of neurons at vulnerable spots in the brain such as thehippocampus and cortex region [150,151,152]. Hence, to be a potential candidate for Alzheimer’s therapy, a compound must pass the blood-brain barrier (BBB) and should be sufficiently bioavailable in the affected area of the brain. The lipophilic nature and low polarity of sesquiterpenes render them bioavailable at specific sites [105,153]. Moreover, the various tools (such as Molinspiration, Swiss ADME) from the molecular modeling docking portal can be employed to predict the probability of a novel compound for crossing the BBB. The ability of the ambrosin, a sesquiterpene, was successfully predicted by using this technique [144]. Moreover, the non-polar solvents can be used for better extraction of lipophilic sesquiterpenes from the herbs. 

Recent clinical studies have shown that the AChE inhibitors significantly alleviate Alzheimer’s symptoms and also lessen the rates of related mortality [154]. However, most of the time the patients discontinue their use due to associated side effects. Hence, it is very important to assess the toxicological profile of the novel compounds. Unfortunately, most of the plant actives reported in this article were not evaluated for the unwanted side effects. Furthermore, the Galliardin, a guainolide-type sesquiterpene, possesses good AChE inhibitory potential, but it also has significant cytotoxic properties [123]. Therefore, it is suggested that the derivatization of potential compounds could be carried out in the future to obtain clinically effective AChE inhibitors with less or no cytotoxicity. Moreover, the promising sesquiterpenes should also be assessed for clinical data to obtainreliable and safe anti-Alzheimer candidates. 

Furthermore, it has been established that for the proper functioning of neurons, there should be a critical balance between the excitatory and inhibitory neurotransmitters [155,156,157,158]. It is evident that β-amyloid (Aβ) could also enhance the translation of GABA_A_ receptor (at α_6_ subunit) by the phosphorylation of mTOR and ERK in the cerebellum of experimental mice [28,159,160], and hence an increase in inhibitory transmission at synapse affects the learning and memory process [161]. Sesquiterpenes such asBilabolide could help in achieving the equilibrium of excitatory and inhibitory transmission because it was reported as an antagonist to α_1_β_2_γ_21_ subunits of GABA_A_ receptors that enhanced the excitability of neurons in hippocampal slices [139]. Moreover, the Aβ application also increases the concentration of AChE in cultures and the simultaneous feeding of experimental animals with farnesene (a sesquiterpene) significantly reduces the enzyme concentration [162]. Hence, it could be interesting to explore the effect of potential AChE inhibitors in β-amyloid deposition and in GABAergic neuronal transmission.

In recent years, the treatment paradigm for AD is moving toward alleviating the inflammation associated with the development of this disease [163,164]. Hence, the exploration of novel sesquiterpenes for the expression of NF-κβp65 could be a target for scientists for the management of neurodegenerative disorderssuch asAD.

Thus, this review paper comprehensively discussed the various types of sesquiterpenes, active constituents related, and their devoted involvement toward the improvement of cholinergic transmission by inhibiting the AChE. Their involvement in different alternate targets for AD management was also discussed briefly. The described sesquiterpenes are supposed to act as novel compounds for the researchers willing to find some other alternatives than the existing ones for the possible treatment of neurodegenerative diseases, i.e., Alzheimer’s disease.

## 6. Conclusions

No doubt extensive researchhas been carried out to explore suitable candidates for AD management. In spite of the availability of bulk knowledge related to this progressive disorder, we have access only to symptomatic treatment, not to a complete cure. Improvement in cholinergic transmission by inhibiting the AChE was considered to delay the AD progression and relieve the AD-associated symptoms. This review mainly focuses on the various sesquiterpenes acting as potential acetylcholinesterase inhibitors and also summarizes their role on alternate AD targets. The literature revealed the potential of sesquiterpenes such as amberboin and lipidiol in the inhibition of AChE. Moreover, the sesquiterpenes such as Farnesene, Bilabolide, and Jatamansone were found to exert a significant effect on different AD targets such as Aβ plaque, neuron excitability, and oxidative stress. Nowadays, the paradigm for AD management has shifted toward the management of neuroinflammation by novel compounds. Sesquiterpenes such asambrosin and nootkatone alleviate neuroinflammation by reducing the expression of transcription factor NF-κβp65.

In future studies, a scientific approach can be acquired from this systematized information to prepare a baseline for the further consideration of natural cognitive enhancers (especially sesquiterpenes) in age-related diseases such asAD. Moreover, the described moieties could be further investigated for better derivatives in terms of safety and efficacy. Furthermore, it is also speculated that a mere delay in the symptoms of AD by one year could reduce the patient load significantly (upto 9.2 million) in 2050. Hence, it can also be concluded that the use of suggested herbs as nutraceuticals/dietary supplements could significantly alleviate the symptoms of AD and prove highly beneficial for mankind.

## Figures and Tables

**Table 1:** Some Sesquiterpenoids that haveanticholinesterase inhibitory activity.

Name of Plant	Part Used (Family)	Solvent/Method Used for Extraction	Extract/Volatile oil/Phytoconstituents	Acetyl-Cholinesterase Inhibitory/Anti-Alzheimer Potential	Ref.
*Lycopodiastrum casuarinoides*	Whole plants (Lycopodiaceae)	EtOH (75%)	7, 9 -diene -1,4 -epoxy-2 -hydroxy -10 -carboxylic acid [13]	Potential inhibitory effects against AChE and BuChE with an IC_50_ = 9 ± 1 μM and 9 ± 1 μM respectively	[98]
*Lycium europaeum* Linn.	Roots and leaves (Solanaceae)	Ethanol	Ethanolic fraction	Ethanolic fraction of leaves (at 15 mg/kg) exhibited effect on learning and memory of experimental animals with IC_50 =_ 76 ± 2 mg/mL	[99]
*Aquilaria or Gyrinops*	Roots and resinous heartwood (Thymelaeaceae)	95% EtOH	2-oxoguaia-1(10),3,5,7(**11**), 8-pentaen-12,8-olide (**14**) (Sesquiterpenoids) 4β,7α-*H*-eremophil-9(**10**)-ene-12,13-diol (**15**) (Eremophilane- sesquiterpenoid) 4β,5α,7α,8α-*H*-3β-hydroxy-1(10)-ene-8,12-epoxy-guaia-12-one (**16**) (−)Gweicurculactone (**17**) (Guaiane-sesquiterpenoid)	The isolated compounds showed the AChE inhibitory activity at 50 μg/mL ((IC_50_) IC_50_ (14) = 226 μM IC_50_ (15) = 140 μM IC_50_ (16) = 141 μM IC_50_ (17) = 202 μM Tacrine ( + ve control) = 65 ± 1 μg/mL	[100]
*Valeriana jatamansi* Jones	Roots and rhizomes (Valerianaceae)	EtOH (95%)	Valeriananoids D (**18**) Valeriananoids E (**19**)	At the conc. of 50 μM showed AChE inhibition potential activity	[101]
*Aquilaria sinensis (Lour.)*	(Thymelaeaceae)	EtOH (95%)	3-oxo-7-hydroxyl holosericin A (**20**) 1,5;8,12-diepoxy-guaia-12-one (**21**) 8αβ)-octahydro-7-[1-(hydroxymethyl (**22**) 7α H- ethenyl]-1,8α-dimethyl naphthalen-4α(2*H*)-o (**23**) Neopetasane (**24**)	Moderate inhibitory activities against Acetylcholinesterase (AChE) at 50 µg/mL, IC_50_ (20) = 75 μM IC_50_ (21) = 53 μM IC_50_ (22) = 71 μM IC_50_ (23) = 87 μM IC_50_ (24) = 324 μM	[102]
*Rhodomyrtus tomentosa*	leaves and stems (Myrtaceae)	Petroleum ether (PE) extract	(Triketone-sesquiterpene meroterpenoids)	AChE inhibition rate is 81% at 500 μg/mL)	[103]
*Nelumbo nucifera*	Seeds (*Nelumbonaceae)*	essential oil (EO), crude extract, and subsequent fractions	Essential oil mainly comprised of oxygenated mono and sesquiterpenes	The ethyl acetate fraction and EO caused significant inhibition of acetyl-cholinesterase and butyryl-cholinesterase with IC_50_ = 70 ± 1, 64 ± 1 and 75 ± 0.3, 58 ± 0.2, respectively in a dose-dependent manner. EO was found to be non-competitive inhibitor of AChE.	[104]
*Asteriscus maritimus* (Linn.) Less	Different parts of the plant (Asteraceae)	Hydrodistillation	Oxygenated sesquiterpenes of Essential oil	Among the essential oils obtained from flowers, leaves, and stems, the flower oil was found to exhibit the highest anti-acetylcholinesterase activity (IC_50_ = 95 µg/mL)	[105]
*Myrciaria floribunda* (H. West ex Willd.)	Essential oil of fruit peel (Myrtaceae)	Hydrodistillation method	Essential oil with different sesquiterpenes	Essential oil exhibited the AChE inhibitory potential with IC_50_ = (0.08 μg/mL and 23 μg/mL). Neostigmine (as the standard used) had an IC_50_ of 23 μg/mL and 6 μg/mL).	[97]
*Nigrospora oryzae and* *Irpex lacteus* (Fungus on plant )	Culture	Acetone	Tremulanesesquiterpenes	AChE inhibitory potential concentration of 50 μM.	[106]
Curry Leaf	*Murraya koenigii* (Rutaceae)	Hexane and methanol	Essential oil	Inhibitory activities of active compounds of curry leaves against β-secretase were found and hence reported to be helpful in preventing dementia (AD). methanolic extract (70%) also showed weak inhibitory activity at 500 μg/mL against AChE	[107]
*Teucrium persicum* Boiss	Aerial parts (Labiatae)	Methanol (85%)	Guaiasistanol (**25**) (Guaianesesquiterpenoid)	Moderate inhibition of AChE (28%) by the isolated compound.	[108]
*Daphne holosericea* (Diels) Hamaya	Dry stems (Thymelaeaceae)	Extracted with EtOH (95%) under reflux three times	Holosericin B (**26**) (Guaiane Sesquiterpenoids)	The isolated compound showed a moderateAChE Inhibitory Activity with 31% inhibition.	[109]
*Aquilaria sinensis* (Lour.) Gilg	Heartwood (Thymelaeaceae)	Refluxed with Ethanol (95%)	Extract	EtOAc extract showed weak AChE inhibitory activity	[110]
*Homalomena sagittifolia*	Rhizomes (Araceae)	Macerated with aqueous methanol	1α,4β,7β- eudesmanetriol (**27**) 1β, 4β, 7β-eudesmanetriol (**28**) (Sesquiterpenoids)	Inhibition of acetylcholinesterase with IC_50_ (I) = 26 ± 4; (II) = 250 ± 8 μM	[111]
*Valeriana officinalis*	Roots (Caprifoliaceae)	EtOH (95%)	Spatulenol (**29**) (Sesquiterpenoids)	AChE was inhibited at 100 mM (49%)	[112]
*Marsupella alpine* (Chinese liverwort)	Whole plants (Gymnomitriaceae)	95% EtOH (95%)	Marsupellin A (**30**) Marsupellin B (**31**) (ent-Longipinane-Type Sesquiterpenoids)	A bioautographic TLC assayforAChE inhibition was performed and compound showed moderate inhibition at 5 μM (28% and 26% respectively).	[113]
*Santalum album*	Heartwood (Santalaceae)	Steam distillation	α-santalol (**32**), Sandalwood oil (rich in sesquiterpenoid alcohols) the major constituent of the oil	TLC-bioautographic and colorimetric methods are used. Essential oil is found to be a potent inhibitor of tyrosinase IC_50_ = 171 µg/mL) and cholinesterase IC_50_ = 5–58 µg/mL. For α-santalol, AChE Inhibition Zone (mm^2^) and BChE Inhibition Zone (mm^2^) were reported as 326 ± 19 and 425 ± 27 respectively.	[114]
*Hedychium gardnerianum* Sheppard ex Ker-Gawl	Leaf essential oil (Zingiberaceae)	Hydrodistillation	Sesquiterpene hydrocarbons (47.8 to 52.7%) and oxygenated sesquiterpenes (15.2 to 16.3%) are main constituents of oil	Microplate Assay was performed and the strongest inhibition against AChE was displayed by the sample collected from Furnas (FU) at IC_50_ = 1 mg/mL.	[115]

**Table 2:** Marketed formulation of the sesquiterpenes used for memory-enhancing activity.

Phytoconstituents	Marketed Formulation	Dose and Form	Manufacturing Company
Huperzine A	Huperzine A Dietary supplements	200 MCG 120 tablets	National INC., P.O 2118, Santa Cruz CA 95062.
Huperzine-A	Huperzine Rx Brain ^R^	50 MCG	National Organics Lab. INC. Nature Plus, USA
Bilobalide	HAVASU NUTRITION Neuro IGNITE	Capsule *Ginkgo biloba* extract 50 mg Huperzine A (*Huperzia serrata* extract/leaf) 10 mcg	Havasu Nutrition, LLC19,046Bruce B, Downs Blvd#1090, Tampa, FL 33647
Bilobalide	Healthy Hey Ginseng with Ginkgo Extract Support memory and concentration	160 MG capsule *Ginkgo biloba* 60 mg + *Ginseg panax* 100 mg	Healthy Hey foods LLP. 227, Building No-58, Mittal Ind, Estate Andheri (E), Mumbai, 400059.
Bilobalide	Vitamin *Ginkgo biloba* (for brain support)	500 mg Capsule	Plot No-57/1, Phase -1, G.I.D.C, vapi, Gujrat-396 195, India
Bilobalide	*Ginkgo biloba*	500 mg *Ginkgo biloba* 120 mg *Bacopa monnieri* extract 380 mg	Herbal farm Lifecare Pvt. Ltd., C-86, Pocket C, 2^nd^ Floor, Okhla Industrial Area, Phase-I, New Delhi-110020.
Bilobalide	Natures velvet *Ginkgo Biloba*	Capsule, 80 mg	Natures Velvet Lifecare, 103, Liberty Plaza, himayat Nagar, Hyderabad, Pin-500029, Telangana, India.
Bilobalide	Simply Nutra *Ginkgo Biloba* with Brahmi	Capsule, 500 mg 120 mg + 380 mg,	Soulager Healthcare Private Limited., Scheme 53, Plot No-100, India, M.P 452010.
Bilobalide	*Ginkgo biloba*	Capsule (60 mg)	Sanathal Ring road, Opp GEB station, Sanathal Ahmedabad, Gujarat.
Bilobalide	Nutriosys *Ginkgo biloba*	Capsule (360 mg)	Sanathal Ring road, Opp GEB station, Sanathal Ahmedabad, Gujrat.
Bilobalide	iAYUR *Ginkgo Biloba*	Capsule (500 m)	Suimabhan Commerce Private Limited A-1/224, Janakpuri, New Delhi 110058
Bilobalide	Vita green *Ginkgo biloba*	Capsule, 500 mg	Green cross, health Innovation, Plot No-57/1, Phase 1, GIDC, Vapi -396195.
Bilobalide	CoreFX Labs	Capsule *Ginkgo biloba* leaf (24% extract) 50 mg, *Bacopa monnieri* leaf extract (20% bacosides ) 120 mg, Huperzine A (aerial plant) 10 mcg	Xtreme Ai, 100 Orandorf, Dr# 775, Brighton MI 48116.
Bilobalide	Body BRAIN SUPPORT Dietary Supplements	Capsule *Bacopa monnieri* whole plant extract 200 mg, *Ginkgo biloba* leaf extract 100mg, Huperzine A ( *Huperiza serrata* leaf standard extract) 250 mcg and others	1 Body 5940 S. Rainbow Blvd, Las Vegas, NV 89118
Huperzine A	FOCUS ELITE Support Brain’s Focus, Memory and clarity	Capsule Huperzine A complex ( *Huperzia serrata* 25 mcg), *Ginkgo biloba* leaf extract 50 mg, *Bacopa monneri* extract of whole herb 75 mg and others	Elite source labs 130 Corridor Rd,# 3259, Ponte Vedra, FL 32004, USA
Bilobalide	NOW Brain Elevate Cognitive functions With *Ginkgo Biloba*, Rose OX and Phosphatidyl Serine	Capsule Ginkgo Extract (*Ginkgo biloba* leaf ) 60 mg, Huperzine complex ( *Huperzia serrata*/Moss) 25 mcg and others	NOW FOODS, 395 S Glen Ellyn Rd, Bloomingdale, IL 60108, USA
Bilobalide	NEURA–SPARK	Capsule, *Ginkgo biloba* 50 mg, Huperzine A 10 mcg, *Bacopa monnieri* ( 20% bacosides, herb) 300 mcg and others	NUTRACHAMPS Inc. AURORA, ON, L4G1M2
Bilobalide	Vitacern BRAIN FUEL	Capsule *Ginkgo Biloba* leaf ( 24% extract) 50 mg, *Bacopa monnieri* leaf extract 120 mg, Huperzine A ( aerial plant ) 10 mcg and others	Vitacerna, Suite #7004, 3422 SW, 15 street, Deerfield Beach, FL33,442USA
Huperzine –A	Double Woods Supplements HUPERZINE –A	Tablet Huperzine-A 200 mcg	Double Woods LLC, 3510 SCOOTS LN STE 219, PHILADELPHIA, PA 19129, United States
Jatamansone Celastrine	Ayukriti HERBALS Memokriti ^R^ capsule	Brahmi (*Bacopa monnieri* ) Pl Ext. 100 mg, Jatamansi (*Nardostachys jatamansi*) Rt. 60 mg. Jyotishmati (*Celastrus paniculatus*) Rt. 60 mg and others.	HARASHA PHARMA Private Limited. PiyauManihariNarela road, Kundli, Distt. Sonepat (Haryana) Harasha Pharma Pvt Ltd 159 A DG II D BLOCK VIKASPURI NEW DELHI
Jatamansone	Indiveda Ayurvedic Herbs Organic Jatamansi Root Powder (*Nardostatchysjatamansi*)	100 g powder, Pure organic Jatamansi Root Powder	Ayuish Biotech &lifescience Company, Chanarthal road, Kurukshetra, Haryana-136119
Jatamansone	Ayurvedic Proprietary Medicine Nurayurich Capsule	Capsule *Nardostachys jatamansi* 75 mg each Others 67 mg	Saived Pharma Private Limited, C 4/35, MIDC, Jejuri, Tal-Purandar, Distt-Pune-412303
Jatamansone	VitaGreen Jatamansi	Capsule Jatamansi Extract (*Nardostachys jatamansi*) 500 mg	Manufacturing LicenceNo GA/1736, Green Cross Health Innovation, Plot No- 57/1, Phase 1, G.I.D.C, Vapi-396195.
Jatamansone	HealthVit Jatamansi Powder *Nardostachys jatamansi*	100 g Jatamansi root powder (*Nardostachy s jatamansi*) 100% *w*/*w*	West-Coast Pharmaceutical Works Ltd., Ahmedabad -382 481, Gujrat.
Jatamansone	Himalaya Herbal Health care Mentat DS	100 mL Syurp *Bacopa monnieri* 288 mg, Jatamansi 104 mg, *Celastrus paniculatus* 64 mg	Himalaya Drug company, Makali, Bengaluru
Jatamansone	Herbal Hills Jatamansi Powder *Nardostachys jatamansi*	100 g powder Jatamansi Powder	Isha Agro Developers PVT.LTD. Unit No- 36 A/55AB, LonavalaCo.op. Indl. Est. Ltd, Village- Nangargaon, Lonavala, Taluka-Maval, Distt Pune-410401, Maharashtra, India.
Jatamansone	Amalath Jatamansi Extract	Jatamansi root extract, 5:1 (Capsule 500 mg)	Devki Pharmacy, Kakheri, Kaithal -136033, Haryana, India.
Jatamansone	Kerala aurveda™ GANDHA THAILAM	GANDHA THAILAM Capsule 300 mg Jatamansi (*Nardostachys jatamansi*) 0.5 mg and others	Kerala Ayurveda, Ltd, Athani 683585, Aluva, Kerala, India.
Bilobalide	Standardized *Ginkgo Biloba* Extract as Herbal Supplement	Capsule *Ginkgo Biloba* Extract (leaf) 60 mg (50:1)	21st Century Healthcare, Inc. 2119 S. Wilson St. Tempe, AZ 85282, USA.
Bilobalide	MRM GINKGO B Supports circulation and mental functions Dietary Supplements	Capsule *Ginkgo Biloba* Extract (60 mg)	MRM 2665 Vista Pacific Dr. Oceanside CA 92056, USA
Bilobalide	Ginkgo+Bilbery+Lutein	*Ginkgo Biloba* 60 mg (capsule)	Biotrex Nutraceutical, Sanathal ring Road, Opp GEB Station, Sanathal, Ahmedabad, Gujrat.

## Abbreviations

AChE	Acetylcholinesterase
AD	Alzheimer’s disease
APP	amyloid precursor protein
ARD	Aromadendrane-4β,10α-diol
Aβ	Amyloid Beta
BACE1	Β-site APP Cleaving Enzyme1
BChE	Butylcholinesterase
BuTChERK	ButyrylthiocholineExtracellular signal regulated kinase
FPP	Farnesyl Pyrophosphate
HDS	13-hydroxy-8,9-dehydroshizukanolide
ICO	Isocubebenol
LAMI	Learning and Memory impairment
LPS	Lipopolysaccharide
MDA	Malondialdehyde level
mTOR	Mammalian target of rapamycin
MWM	Morris Water Maze
NF-κB	Nuclear factor kappa-light-chain-enhancer of activated B cells
NLRP3	Nucleotide-binding domain and leucine-rich repeat protein 3
NMDA	N-methyl-D-aspartate
PDA	Podoandin
PS-1	Presenilin-1
SC	Sesquiterpenecoumarins
SLs	Sesquiterpene Lactones
